# Association between estimated glomerular filtration rate and clinical outcomes in ischemic stroke patients with high-grade carotid artery stenosis

**DOI:** 10.1186/s12883-021-02154-3

**Published:** 2021-03-19

**Authors:** Chung-Hao Chao, Chia-Lun Wu, Wen-Yi Huang

**Affiliations:** Department of Neurology, Chang Gung University, College of Medicine, Chang Gung Memorial Hospital, Keelung branch, No.222, Mai-Jin Road, Keelung, 204 Taiwan

**Keywords:** Ischemic stroke, Carotid artery stenosis, Glomerular filtration rate, Mortality, Outcome

## Abstract

**Background:**

Chronic kidney disease has been identified as a risk factor affecting stroke prognosis. High-grade carotid artery stenosis (CAS) is associated with distal hemodynamic compromise. The association between the estimated glomerular filtration rate (eGFR) and ischemic stroke (IS) outcome in patients with high-grade CAS remains unclear. We aimed to investigate the association between eGFR and outcomes of acute IS patients with high-grade CAS.

**Methods:**

From January 1, 2007 to April 30, 2012, we enrolled 372 acute IS patients with high-grade CAS and prospectively observed them for 5 years. The eGFR on admission was assessed using the Modification of Diet in Renal Disease Study equation. Demographic features, vascular risk factors, comorbidities, and outcomes were compared between different eGFR levels.

**Results:**

Among 372 individuals, 76 (20.4%) had an eGFR < 45, 65 (17.5%) had an eGFR between 45 and 59, and 231 (62.1%) had an eGFR ≥60 mL/min/1.73 m^2^. Compared to other groups, in the eGFR < 45 mL/min/1.73 m^2^ group, the prevalence rates of hypertension, diabetes mellitus, coronary artery disease, congestive heart failure, valvular heart disease, and gout were significantly higher (*P* = 0.013, *P* = 0.030, *P* = 0.001, *P* < 0.001, *P* = 0.043, and *P* < 0.001, respectively). Patients with eGFR < 45 mL/min/1.73 m^2^ demonstrated lower hemoglobin and total cholesterol levels compared with other groups (*P* < 0.001 and *P* = 0.048). The blood potassium and uric acid levels were significantly higher in patients with eGFR < 45 mL/min/1.73 m^2^ (*P* < 0.001 and *P* < 0.001). The multivariate Cox proportional hazards model indicated that eGFR < 45 mL/min/1.73 m^2^ was a significant risk factor for 5-year all-cause mortality in IS patients with high-grade CAS after adjusting for these variables (hazard ratio = 2.05; 95% CI = 1.31–3.21; *P* = 0.002).

**Conclusions:**

eGFR < 45 mL/min/1.73 m^2^ was associated with an increased risk of 5-year all-cause mortality in acute IS patients with high-grade CAS. Whether aggressive treatment of chronic kidney disease in IS patients with high-grade CAS can improve stroke outcomes should be confirmed in future studies.

## Background

Carotid artery stenosis (CAS) is a well-recognized cause of cerebral ischemia, and more severe luminal stenosis is associated with distal hemodynamic compromise [1]. Chronic kidney disease (CKD) has been identified as a novel risk factor affecting stroke prognosis, and the estimated glomerular filtration rate (eGFR) is one of the common parameters representing the renal function [2–6]. Although CKD is generally regarded as a strong predictor of mortality and poor outcome in patients with acute stroke [5], the findings of previous studies on the association between eGFR and outcome of ischemic stroke (IS) are inconsistent. Some studies indicate that a reduced eGFR is associated with a higher mortality rate in patients with IS [3, 5, 7], whereas some other studies suggest that a reduced or highly elevated eGFR is associated with a higher mortality rate in patients with IS [2, 4].

Patients with severe CAS have a higher risk of stroke and mortality [8], and carotid artery revascularization is capable of reducing the stroke risk [9, 10]. Patients with CKD are at increased risk of cardiovascular disease and the progression of atherosclerosis [11, 12]. As atherosclerosis is a systemic condition and the prevalence of severe CAS increases with the prevalence of coronary artery stenosis [13], it would be important to understand the association between eGFR and stroke outcome in patients with high-grade CAS. To our knowledge, the association between the level of eGFR and stroke outcome in patients with acute IS has not been assessed specifically in patients with high-grade CAS (≥ 70%) [14].

As a result, the aims of this study were to investigate: (1) the clinical characteristics of acute IS patients with high-grade CAS, comparing patients with different ranges of eGFR and (2) the association between the eGFR level and 5-year outcomes in acute IS patients with high-grade CAS.

## Methods

### Ethical standards

This clinical study followed the Declaration of Helsinki and was approved by the Medical Ethics Committee of Chang Gung Memorial Hospital (CGMH), Taipei, Taiwan (IRB 201800689B0). As this was an observational study without active intervention, and all subjects were diagnosed, treated and followed as clinical routines, the Medical Ethics Committee of CGMH, Taipei, Taiwan waived the requirement for written informed consent.

### Study population

All study patients with acute first-ever IS and high-grade CAS were recruited from the Stroke Unit of the Department of Neurology in Keelung CGMH from January 1, 2007 to April 30, 2012. The diagnosis of acute IS was made in accordance with the World Health Organization criteria, and was confirmed by brain magnetic resonance imaging or computed tomography (CT) scans [15]. The definition of high-grade CAS was ≥70% stenosis at the carotid artery [14]. To evaluate the severity of CAS, carotid duplex ultrasound was performed within 7 days after stroke onset. Duplex examinations were performed using Philips EnVisor C (Philips Medical Systems, Nederland B.V.) with a 7-MHz linear-array probe by an experienced technical assistant who was supervised by 2 of the authors. The diameter of the residual lumen at most stenotic portion (d) and the external diameter of the artery at the same level (D) were measured, and the degree of stenosis was calculated using the following equation: percentage of stenosis = (D – d) 100/D [14, 16].

If the ultrasound results revealed ≥70% stenosis at the carotid artery, brain magnetic resonance angiography was arranged to confirm the CAS and evaluate the location and volume of the infarction. We only recruited patients with high-grade CAS with a degree of stenosis ranging from 70 to 100% [14], and acute stroke symptoms can be correlated with the vascular territory of the carotid artery with CAS. The exclusion criteria were as follows: (1) the location of infarction cannot be correlated with the vascular territory of high-grade CAS; (2) patients received revascularization treatments; (3) patients with previous IS, cerebral hemorrhage, or stroke of uncertain causes; and (4) patients with severe medical diseases, such as malignancy, liver cirrhosis, or end-stage renal disease with hemodialysis or peritoneal dialysis.

The renal function of the recruited patients with acute IS and high-grade CAS was assessed using the Modification of Diet in Renal Disease Study equation for eGFR: eGFR (mL/min/1.73 m^2^) = 186 × (serum creatinine [mg/dL])^-1.154^ × (age [years])^-0.203^ × (0.742 if female) [17].

All recruited patients were stratified by eGFR into the following three groups for statistical analysis: (1) eGFR < 45 mL/min/1.73 m^2^; (2) eGFR 45–59 mL/min/1.73 m^2^; and (3) eGFR ≥60 mL/min/1.73 m^2^. We grouped the recruited patients according to the stages of chronic kidney disease [18]. eGFR ≥60 mL/min/1.73 m^2^ indicated CKD stage 1 (normal kidney function) and stage 2 (mild loss of kidney function); eGFR 45–59 mL/min/1.73 m^2^ indicated CKD stage 3a (mild to moderate loss of kidney function); eGFR < 45 mL/min/1.73 m^2^ indicated CKD stage 3b (moderate to severe loss of kidney function), stage 4 (severe loss of kidney function), and stage 5 (kidney failure).

### Clinical assessments

Comorbidities and vascular risk factors such as hypertension, diabetes mellitus (DM), hyperlipidemia, smoking, atrial fibrillation (AF), valvular heart disease, peripheral arterial diseases, and coronary artery disease (CAD), were identified after an in-depth review of the medical records. Hypertension was diagnosed as systolic blood pressure > 160 mmHg and/or diastolic blood pressure > 95 mmHg on two different occasions with the first measurement within 2 days after stroke onset and the second measurement taken more than 5 days after the stroke, or known hypertension was diagnosed by a clinician [19, 20]. DM was identified in patients with fasting plasma glucose ≥7.0 mmol/L or a 2-h value in the oral glucose tolerance test ≥11.1 mmol/L or a random plasma glucose concentration ≥ 11.1 mmol/L in the presence of symptoms or patients with previously treated DM [21]. AF was identified by ECG and/or 24-h ECG monitoring. Cigarette smoking was identified as a current smoker or a smoker with cessation less than 5 years ago. Laboratory assessments, such as complete blood cell count, biochemistry studies, lipid level, glycohemoglobin, coagulation testing, 12-lead electrocardiography, and transthoracic echocardiography, were performed within 1 week after acute stroke onset. The clinical IS subtypes of the Oxfordshire Community Stroke Project classification [22] and the scores of the National Institutes of Health Stroke scale, Modified Rankin scale [23], and Barthel index were recorded.

### Follow-up

The timings of follow-up were at the first and third months after the initial assessment of acute ischemic stroke and then every 3 months. All recruited patients were followed up for 5 years after acute ischemic stroke. The follow-up period was from January 1, 2007 to April 30, 2017. The primary end points included 5-year mortality, stroke-related death, or stroke recurrence. Every cause of death was reviewed. New major medical problems, such as death, recurrent IS, cerebral hemorrhage, cancer, head injury, and reason for rehospitalization, were recorded.

### Statistical analysis

Continuous variables were expressed as medians (interquartile ranges) as they were not normally distributed. Categorical variables were expressed as a number (percentage) [24]. The clinical characteristics of the patients in the different eGFR groups were analyzed using descriptive statistics. The Kruskal-Wallis or chi-square test was used to assess the group differences [24]. Kaplan-Meier survival analysis was used to estimate the cumulative overall survival for patients with different eGFR levels, and the log-rank test was used to assess the group differences [25]. The Cox proportional hazards model was used to determine the significance of each variable in predicting 5-year mortality. A univariate Cox model was used initially to measure the hazard ratio of all previously identified variables for mortality. Then, a backward, stepwise multivariate Cox regression model was used to identify the risk factors for 5-year mortality [25]. All statistical analyses were performed using IBM SPSS statistics 19 for Windows.

## Results

### Demographic characteristics among IS patients with high-grade CAS

**Fig. 1:**
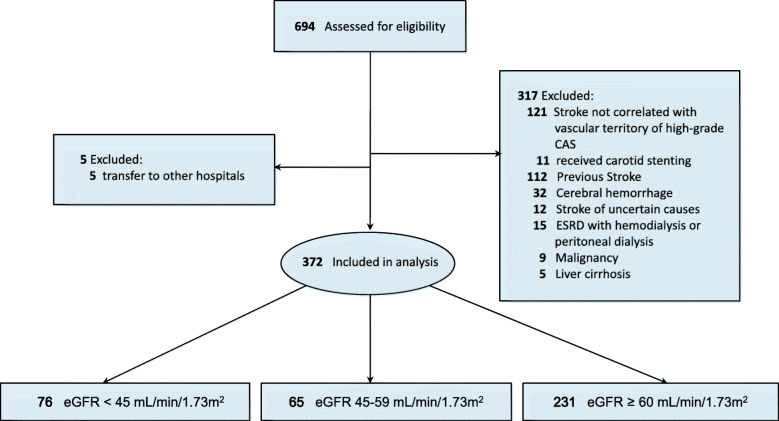
Title: Study Flowchart of patient selection**.** Legends: CAS: carotid artery stenosis; ESRD: end-stage renal disease; eGFR: estimated Glomerular filtration rate

Between January 1, 2007 and April 30, 2012, 694 acute IS patients with high-grade CAS were identified. Among these patients, 322 patients were excluded because they did not fulfill the inclusion criteria. Eleven patients received carotid artery stenting, and these patients were excluded from the study. Finally, a total of 372 acute IS patients with high-grade CAS were enrolled in this study (Fig. 1). The patients were then categorized by the eGFR value as follows: eGFR < 45 mL/min/1.73 m^2^ (76 subjects); 45–59 mL/min/1.73 m^2^ (65 subjects); and ≥ 60 mL/min/1.73 m^2^ (231 subjects). The median age was 73 (63–81) years. Patient characteristics are presented in Table 1. Compared to other groups, in the eGFR < 45 mL/min/1.73 m^2^ group, the prevalence rates of hypertension, DM, CAD, congestive heart failure, valvular heart disease, and gout were significantly higher (*P* = 0.013, *P* = 0.030, *P* = 0.001, *P* < 0.001, *P* = 0.043, and *P* < 0.001, respectively).

**Table 1:** Demographic features of ischemic stroke patients with high-grade carotid artery stenosis

	eGFR (mL/min/1.73 m^2^)			
	< 45 (*n* = 76)	45–59 (*n* = 65)	≥ 60 (*n* = 231)	*P*
Age (years)	77 (71–82)	75 (70–81)	70 (61–79)	< 0.001*
Male (%)	48 (63.2)	44 (67.7)	181 (78.4)	0.018†
Risk factors				
Hypertension (%)	72 (94.7)	53 (81.5)	186 (80.5)	0.013†
Diabetes mellitus (%)	46 (60.5)	29 (44.6)	100 (43.3)	0.030†
Smoking (%)	45 (59.2)	40 (61.5)	161 (69.7)	0.170
Hyperlipidemia (%)	43 (56.6)	43 (66.2)	143 (61.9)	0.500
Atrial fibrillation (%)	18 (24.0)	11 (16.9)	35 (15.2)	0.211
CAD (%)	26 (34.2)	15 (23.1)	34 (14.7)	0.001†
CHF (%)	20 (26.3)	8 (12.3)	13 (5.6)	< 0.001†
Valvular heart disease (%)	4 (5.3)	3 (4.6)	2 (0.9)	0.043†
Peripheral vascular disease (%)	3 (3.9)	2 (3.1)	4 (1.7)	0.513
Gout (%)	34 (44.7)	17 (26.2)	28 (12.1)	< 0.001†
Clinical syndromes				
TACI (%)	13 (17.1)	14 (21.5)	44 (19.0)	0.800
PACI (%)	31 (40.8)	25 (38.5)	87 (37.7)	0.889
LACI (%)	28 (36.8)	20 (30.8)	80 (34.8)	0.743
Laboratory data				
Hemoglobin (mmol/L)	7.63 (6.83–8.32)	8.32 (7.63–9)	8.63 (7.88–9.37)	< 0.001*
WBC (10^9^/ L)	7.60 (6.20–9.78)	7.60 (5.95–9.45)	7.90 (6.30–9.50)	0.670
Platelet (10^9^/ L)	204 (167–268)	193 (160–261)	208 (175–255)	0.604
Glycohemoglobin (%)	6.3 (5.8–7.3)	6.1 (5.8–7.0)	6.0 (5.7–6.9)	0.279
hs-CRP (nmol/L)	57.1 (27.6–160)	47.2 (28.6–91.4)	42.9 (21.0–85.7)	0.081
Total cholesterol (mmol/L)	4.60 (3.62–5.48)	4.81 (4.27–6)	4.89 (4.29–5.56)	0.048*
Na (mmol/L)	138 (136–140)	138 (136–140)	138 (136–140)	0.710
K (mmol/L)	4.1 (3.8–4.6)	3.9 (3.6–4.2)	3.9 (3.6–4.1)	< 0.001*
Uric acid (mmol/L)	0.41 (0.36–0.48)	0.35 (0.29–0.40)	0.34 (0.28–0.39)	< 0.001*
Antithrombotic treatment				
Aspirin (%)	41 (53.9)	47 (72.3)	159 (68.8)	0.032†
Clopidogrel (%)	26 (34.2)	11 (16.9)	54 (23.4)	0.048†
Anticoagulant (%)	9 (11.8)	9 (13.8)	30 (13.0)	0.938
None (%)	3 (3.9)	2 (3.1)	2 (0.9)	0.032†

eGFR indicates estimated Glomerular filtration rate; *CAD* coronary artery disease; *CHF* congestive heart failure; *TACI* total anterior circulation infarcts; *PACI* partial anterior circulation infarcts; *LACI* lacunar infarcts; *WBC* white blood cells; *hs-CRP* high-sensitivity C-reactive protein

Data are presented as median (interquartile range) or absolute numbers (percentage)

*P < 0.05, Kruskal-Wallis test; †*P* < 0.05, Chi-square test

### Clinical course among IS patients with high-grade CAS

Patient characteristics and clinical course are summarized in Table 2. The mean length of acute ward stay and the occurrences of initial impaired consciousness, stroke evolution, in-hospital complications, stroke recurrence, and stroke-related death did not differ between the groups with different eGFRs in IS patients with high-grade CAS. However, the 5-year all-cause mortality rate was significantly higher in the eGFR < 45 mL/min/1.73 m^2^ group compared with other groups (*P* = 0.001).

**Table 2:** Clinical courses of ischemic stroke patients with high-grade carotid artery stenosis

	eGFR (mL/min/1.73 m^2^)			
	< 45 (n = 76)	45–59 (n = 65)	≥ 60 (n = 231)	*P* value
Mean length of stay in the acute medicine ward (days)	11 (7–16)	12 (8–18)	11 (7–17)	0.592
Initial impaired consciousness (%)	16 (21.1)	16 (24.6)	50 (21.6)	0.854
Course of acute stroke stage				
In evolution (%)	16 (21.1)	11 (16.9)	41 (17.7)	0.773
Complications				
Pneumonia (%)	9 (11.8)	12 (18.8)	29 (12.6)	0.393
Gastrointestinal bleeding (%)	18 (23.7)	10 (15.6)	38 (16.5)	0.323
Urinary tract infection (%)	12 (15.8)	9 (14.1)	23 (10.0)	0.330
Glasgow coma scale score				
Upon admission	15 (15–15)	15 (15–15)	15 (15–15)	0.842
Upon discharge	15 (15–15)	15 (14–15)	15 (15–15)	0.777
National Institutes of Health Stroke Scale score				
Upon admission	6 (3–10)	6 (3–12)	5 (3–9)	0.399
Upon discharge	6 (2.-10)	7 (3–11)	5 (2–9)	0.198
Modified Rankin Scale score				
Upon admission	3 (2–4)	3 (2–4)	3 (2–4)	0.198
Upon discharge	3 (2–4)	3 (2–4)	2 (1–4)	0.093
Barthel index score				
Upon admission	60 (40–80)	70 (35–80)	75 (40–90)	0.220
Upon discharge	70 (40–90)	65 (30–90)	80 (40–95)	0.058
Stroke recurrence (%)	18 (23.7)	22 (33.8)	71 (30.7)	0.375
Stroke-related death (%)	3 (3.9)	2 (3.1)	4 (1.7)	0.712
Death (%)	32 (42.1)	18 (27.7)	48 (20.8)	0.001†

eGFR indicates estimated Glomerular filtration rate

Data are presented as median (interquartile range) or absolute numbers (percentage)

*P < 0.05, Kruskal-Wallis test; †*P* < 0.05, Chi-square test

### Survival analysis of mortality in IS patients with high-grade CAS

**Fig. 2:**
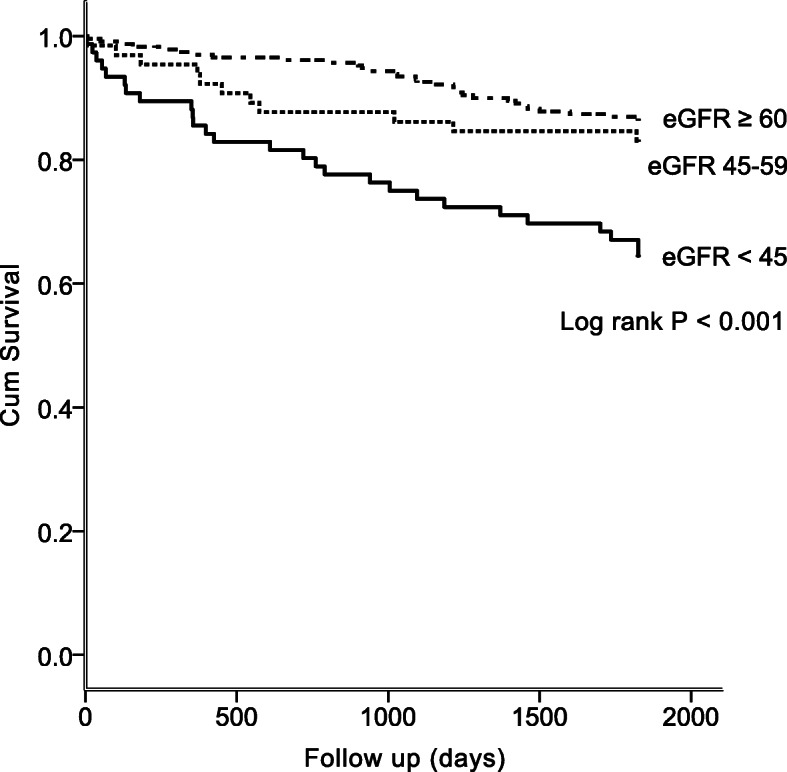
Title: Kaplan-Meier estimates of patient survival during the 5-year study period Legends: Log-rank test, *P* < 0.001. eGFR: estimated Glomerular filtration rate

At the end of the 5-year observation period, 98 patients had died (98/372 = 26.3%), including 32 (42.1%) patients with eGFR < 45 mL/min/1.73 m^2^, 18 (27.7%) patients with eGFR between 45 and 59 mL/min/1.73 m^2^, and 48 (20.8%) patients with eGFR ≥60 mL/min/1.73 m^2^. The cause of patient death in the group with eGFR < 45 mL/min/1.73 m^2^ included acute stroke-related (2 patients), pneumonia and respiratory failure (7 patients), septic shock (9 patients), cardiovascular disease (7 patients), out-of-hospital cardiac arrest (2 patients), end-stage renal disease (2 patients), recurrent large infarction with brainstem compression (1 patient), and unknown causes (2 patients). The Kaplan-Meier survival analysis showed that the eGFR < 45 mL/min/1.73 m^2^ group had a higher mortality rate than the other groups (log-rank test, *P* < 0.001), as shown in Fig. 2.

### Determinants of mortality in IS patients with high-grade CAS

The univariate Cox regression indicated that older age, eGFR < 45 mL/min/1.73 m^2^, lower lipid level, CAD, AF, congestive heart failure, stroke presenting as total anterior circulation infarcts, pneumonia, and gastrointestinal bleeding were potential risk factors (*P* < 0.1) for 5-year all-cause mortality. These variables (*P* < 0.1) were then entered into the multivariate Cox regression analysis. The multivariate Cox proportional hazards model indicated that eGFR < 45 mL/min/1.73 m^2^ was a significant risk factor for 5-year all-cause mortality in IS patients with high-grade CAS after adjusting for these variables (HR = 2.05; 95% CI = 1.31–3.21; *P* = 0.002) (Table 3).

**Table 3:** Cox regression analysis of patient survival during the 5-year study period

	Univariate Cox regression		Multivariate Cox regression	
	HR (95% CI)	*P* value	HR (95% CI)	*P* value
Age	1.06 (1.03–1.08)	< 0.001*	1.03 (1.01–1.05)	0.001†
Male	0.74 (0.45–1.23)	0.242		
eGFR < 45 mL/min/1.73 m^2^	2.90 (1.79–4.70)	< 0.001*	2.05 (1.31–3.21)	0.002†
Hypertension	1.37 (0.68–2.76)	0.379		
Diabetes mellitus	0.90 (0.56–1.44)	0.651		
Hyperlipidemia	0.52 (0.32–0.83)	0.007*		
Coronary artery disease	2.54 (1.55–4.14)	< 0.001*	1.98 (1.28–3.06)	0.002†
Atrial fibrillation	1.89 (1.10–3.23)	0.021*		
Congestive heart failure	2.72 (1.56–4.76)	< 0.001*		
Smoking	1.01 (0.61–1.66)	0.980		
TACI	3.34 (2.06–5.42)	< 0.001*	2.72 (1.75–4.25)	< 0.001†
Pneumonia	2.54 (1.47–4.40)	0.001*		
Gastrointestinal bleeding	2.36 (1.41–3.95)	0.001*	1.73 (1.10–2.71)	0.018†

HR indicates hazard ratio; *CI* confidence interval; *TACI* total anterior circulation infarcts

*P < 0.1 for the univariate Cox regression, and †*P* < 0.05 for the multivariate Cox regression

## Discussion

As noted in previous limited studies about the influence of eGFR level on high-grade CAS, it is important to identify the association between eGFR and clinical outcomes in IS patients with high-grade CAS. We demonstrated that a lower eGFR, especially eGFR < 45 mL/min/1.73 m^2^, was associated with 5-year all-cause mortality. This association remained significant even after adjusting for the established clinical predictors of adverse outcomes.

Previous studies have revealed that impaired eGFR is independently associated with an increased risk of all-cause mortality in the overall population [26, 27] and IS patients [28]. Of the IS patients with high-grade CAS whose eGFR < 45 mL/min/1.73 m^2^ died, only approximately 6% died from acute stroke, which may be explained by the fact that stroke severity was not significantly different between the groups with different eGFRs. A large number (50%) of IS patients with high-grade CAS whose eGFR < 45 mL/min/1.73 m^2^ died from pneumonia with respiratory failure or septic shock.

The classification of CKD based on the level of eGFR had been defined by the guideline from Kidney Disease: Improving Global Outcomes [18]. Previous studies suggested that the impairment of normal innate and adaptive immune systems in CKD predisposes patients to an increased risk of infection, and infection is one of the leading causes of mortality in patients with CKD [29]. Approximately 28% of the patients with eGFR < 45 mL/min/1.73 m^2^ died from cardiovascular disease or out-of-hospital cardiac arrest. CKD is an independent risk factor for cardiovascular disease, and the risk of cardiovascular mortality increases when the condition of CKD deteriorates [30].

Our study revealed that older age, hypertension, DM, CAD and hyperuricemia occurred more frequently in patients with reduced eGFR < 45 mL/min/1.73 m^2^, which was consistent with a previous study [26, 28]. Older age [31], hypertension [32], DM [33], and gout [34] are known risk factors for CKD. The risk of CAD increases as the severity of CKD increases, despite adjustment for traditional cardiovascular risk factors [35]. It is not surprising that the level of hemoglobin was lower in patients with reduced eGFR < 45 mL/min/1.73 m^2^. Decreased erythropoietin production, nutrition deficiency, shortened red blood cell survival and increased iron losses might contribute to the lower hemoglobin levels in patients with reduced eGFR [36].

Recruited patients did not receive revascularization treatment due to the following reasons: (1) some patients hesitated about carotid angioplasty and stenting given concerns about potential complications of the procedure; (2) the vascular surgeons in our hospital were not subspecialized in carotid endarterectomy; (3) some patients could not tolerate long-term dual antiplatelet therapy, such as active peptic ulcers; and (4) some patients had moderate to severe CKD, and they worried that the contrast medium may worsen their renal function.

This study has several limitations. First, we did not identify the pathogenesis of CKD with eGFR < 45 mL/min/1.73 m^2^ in all of the recruited subjects, and different etiologies of CKD may lead to different outcomes [18]. Second, this is an observational study about the association between eGFR levels and 5-year mortality in IS patients with high-grade CAS. Therefore, we cannot conclude that aggressive treatment of CKD in IS patients with high-grade CAS can improve stroke outcomes. However, aggressive control of common risk factors for CKD may provide benefit for reducing long-term mortality in IS patients with high-grade CAS. Third, we did not identify the etiologies of high-grade CAS. Thus, it was difficult to differentiate patients with cardiogenic embolic stroke from those with stroke related to stenosis or occlusion in carotid arteries. Different mechanisms of IS may lead to different outcomes. Fourth, this study was performed in one center, and the sample size was relatively small and may not represent the whole disease group. Fifth, we did not analyze the conditions of other intracranial large arteries and possible collateral flows. The collateral status may be associated with the infarct volume and may influence the stroke outcome [37].

## Conclusions

In acute IS patients with high-grade CAS, eGFR < 45 mL/min/1.73 m^2^ is associated with an increased risk of 5-year all-cause mortality. Whether aggressive treatment of CKD in IS patients with high-grade CAS can improve stroke outcomes should be confirmed in future studies.

## Data Availability

The dataset analyzed during the current study are available from the corresponding author on reasonable request.

## Abbreviations

CAS: Carotid artery stenosis
CKD: Chronic kidney disease
eGFR: Estimated glomerular filtration rate
IS: Ischemic stroke
CGMH: Chang Gung Memorial Hospital
CT: Computed tomography
DM: Diabetes mellitus
AF: Atrial fibrillation
CAD: Coronary artery disease
